# A trainable language model with potential to modulate translation rates in non-model organisms by generating upstream untranslated region sequence libraries

**DOI:** 10.1371/journal.pone.0348455

**Published:** 2026-09-17

**Authors:** Alexander D. Duggan, Matthew P. Newman, David R. McMillen

**Affiliations:** 1 Department of Chemical and Physical Sciences, University of Toronto Mississauga, Mississauga, Ontario, Canada; 2 Department of Cell and Systems Biology, University of Toronto, Toronto, Ontario, Canada; 3 Department of Chemistry, University of Toronto, Toronto, Ontario, Canada; Zhejiang University, CHINA

## Abstract

Tuning protein expression in non-model organisms is often constrained by the lack of validated genetic parts and predictive design tools. Translational tuning through the modulation of upstream untranslated regions (5′-UTRs) offers a potentially organism-agnostic route, but existing methods typically rely on mechanistic assumptions, prior knowledge that may not be available in non-model contexts, or the screening of sequence libraries. Here, we present a simple generative approach for creating synthetic 5′-UTR libraries based solely on the genomic sequence statistics of any desired organism. The method uses a sliding-window n-gram language model applied to native 5′-UTR sequences to produce novel sequences that preserve organism-specific base distributions and motifs without hard-coding specific motifs or mechanistic rules into inflexible statistical templates. We have applied this approach to the model bacterium *Escherichia coli* and the non-model probiotic *Limosilactobacillus reuteri*. Libraries of approximately 1,000 sequences were generated for each organism, from which about 100 unique sequences were experimentally tested for translation of a fluorescent reporter protein. In both organisms, the synthetic libraries yielded a broad range of translation levels from this relatively small number of tested variants. Sequences derived from an organism’s own genomic statistics provided a more uniformly distributed range of translation rates in that organism than sequences derived from the other species. Correlations of individual sequence performance across the two species were weak, and thermodynamic predictions of ribosome binding strength showed very little predictive power, especially in the non-model *L. reuteri*. The results demonstrate that simple statistical language model approaches applied to genomic data can generate functional translational regulatory sequence libraries without detailed mechanistic knowledge or explicit reference to consensus motifs. The approach requires minimal computational resources, avoids reproducing native sequences, and can be readily applied to any organism with a sequenced genome. This strategy may lower technical barriers to expression tuning in non-model organisms.

## Introduction

Tuning the level of protein expression from a cell is a fundamental step in most biological engineering applications: protein expression levels must be tuned to hit desired production rates in bioprocessing; to align with target ligand concentration in biosensing; or to match the required input ranges of other elements in a designed cellular circuit in information processing or control applications. For well-studied organisms such as *Escherichia coli* and *Saccharomyces cerevisiae*, a wide range of methods for tuning protein expression is available, each with known characteristics and an established set of procedures for implementation and troubleshooting; we will call these “model” organisms, following recent usage of that term. Attempts at tuning generally fall into a handful of broad categories [1–4]: (1) transcriptional tuning, in which the promoter of the gene is modified to alter expression; (2) translational tuning, accomplished by modulating the rate at which mRNA transcripts are translated into a protein of interest; and (3) post-translational tuning, in which average functional protein levels are modulated by influencing protein behaviour, for example through the incorporation of peptide tags that alter the degradation or solubility of the protein.

There are compelling reasons to carry out biological engineering in less well-established, non-model organisms: the handful of established usual-suspect organisms represent only a tiny subset of the range of metabolic, genetic, and environmental diversity available in the microbial world, and using the evolved characteristics of an organism can enable access to important properties that are difficult or impossible to reproduce in one of the better established organisms. Non-model organisms may live and function natively in a target environment of interest (such as human or animal intestines, or the soil around plant root systems) and have strong potential to enable the discovery of production pathways for a wide range of compounds through “bioprospecting” and related approaches [5,6].

Working in less well-studied non-model organisms comes at a considerable cost: the standard approaches for tuning protein expression are most often not available. Organisms may not have a known library of transcription factors or transcriptional initiation sequences to be used in transcriptional tuning, they may lack established sets of degradation or solubility peptide tags for post-translational tuning, and protocols and methods may not be sufficiently established to yield consistent results with these methods. This leaves translational tuning as an appealing approach when working in a non-model organism, but this route also often suffers from a lack of validated protocols and/or insufficiently detailed mechanistic information on the regulatory influences involved, which may vary considerably across organisms. Here, we present a simple method of genetic sequence generation and show that it can generate libraries of upstream untranslated regions that exhibit a wide range of translation rates, in both *E. coli* and the non-model organism *Limosilactobacillus reuteri*. *L. reuteri* is a probiotic intestinal bacterium found in a wide range of vertebrate hosts (including humans), with strain-host specific mucosal adhesion adaptations that favour lengthy gut colonisation; these features are of interest for biological engineering efforts aimed at sensing or manipulating the intestinal environment in humans or other species [7–10].

Immediately before the start codon of a gene of interest is an upstream sequence denoted the 5′‑UTR (the UnTranslated Region at the 5′ end of an mRNA transcript), with a functionally relevant length that varies with by organism and genetic context, with eukaryotes generally having longer-range influences than prokaryotes [11,12]; here, we will focus on the 35 bases upstream of the start codon, a sui length for prokaryotic translational regulation. mRNA transcripts are bound to a ribosome through one or more sequences within the 5′‑UTR, with translation into an amino acid chain commencing at the start codon. The details of this ribosomal mRNA capture vary between organisms but generally involve some form of sequence-specific recognition between the mRNA strand and ribosomal RNA (rRNA) sequences incorporated into a ribosomal complex; examples include the Shine-Dalgarno sequence in many bacteria and the family of Kozak sequences in eukaryotes. The process of translational initiation depends in a complex manner on multiple factors, including the secondary structure of the mRNA strand, the nature of the coding sequence itself, and potential influences from the 3′‑UTR downstream of the gene of interest [12,13].

The overall translation rate of a given mRNA transcript is a function of these sequence-dependent factors, with the 5′‑UTR sequence clearly playing a substantial role in the process [12,13]. Efforts have been made to create translation rate models capable of forward prediction (given a sequence, predict its translation rate) and inverse prediction (for a desired translation rate, generate sequences to yield that rate). Methods have varied, including mechanistic representations of the mRNA-ribosome binding and its associated thermodynamic properties [14,15], kinetic models of the translational initiation process [16,17], and data-driven models that use measured translation levels to extract regularities from the associated sequences and thus enable statistical prediction of the translation rates of novel sequences [18–22]. The approaches most similar to the approach we propose here employ genomic sequence information to generate results in a variety of applications, including the identification of genes or other sequences of interest through hidden Markov models or related statistical approaches [23–25] and the generation of statistics-preserving DNA sequences driven by finite genomic contexts [26]. Though some success has been achieved in these modelling efforts, the ability to reliably obtain desired translation rates on demand remains somewhat elusive. If the requirement is generating a specific translation rate, or covering a desired range of translation rates, in an arbitrary organism that may be a non-model species, we are not aware of a clear predictive solution available that reliably operates across both model to non-model organisms.

Characterizing sets of candidate sequences experimentally offers a strong alternative or complement to predictive sequence generation: libraries of 5′‑UTR candidate sequences are synthesized and their translational efficiencies are then measured directly. These approaches have most often been applied in model organisms [13,27–29], but there is no barrier in principle to extending them into non-model organism applications, except perhaps that some screening approaches are based on very large library sizes [21,22], making them challenging to apply without access to substantial infrastructure.

To support our own work in non-model organisms [30,31], we sought to develop a solution with several key properties: (1) it should be as organism-agnostic as possible, allowing the same approach to be applied to any organism (model or non-model); (2) it should not rely on embedded assumptions about the relative influence of portions of the 5′‑UTR on the translation process (since such assumptions may fail to transfer between organisms); (3) it should not require intensive *in cellulo* screening of huge libraries of candidate sequences (which may not be practical or affordable in all research contexts); (4) it should not rely on exact replication of existing sequences, to avoid spontaneous homologous recombination, cross-talk, or other forms of interaction between synthetic and genomic sequences; and (5) it should be implementable without recourse to large amounts of computational power.

The solution we present here combines data-driven and library screening approaches, and satisfies all of our goals: (1) it can be applied to any organism with a known genomic sequence; (2) it incorporates no prior assumptions about specific sequence regions; (3) our tests show that generating on the order of 1000 sequences and testing on the order of 100 of them can yield a wide range of translation levels in two different organisms (one model, one non-model); (4) the sequences generated in our approach have a vanishingly small probability of matching any existing sequence from the genome; and (5) the algorithm is straightforward enough to be readily implemented with commercial desktop/laptop levels of computational power.

## Results and discussion

**Fig 1:**
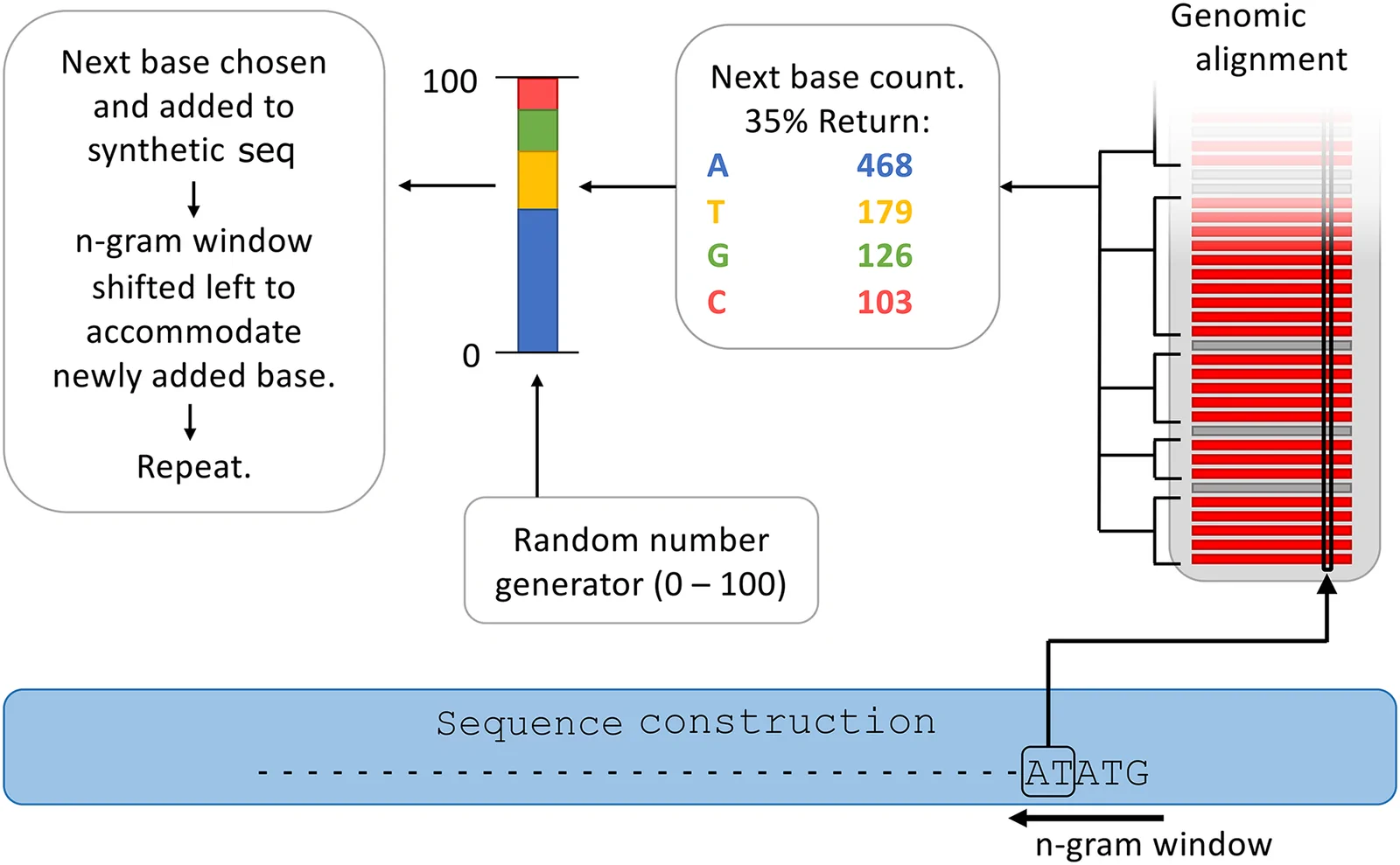
Graphical depiction of the language model. Beginning at the start codon, the algorithm takes the 5′ end of the emerging sequence and queries the genomic 5′ UTR pool for sequences matching the current context. The frequency of each base’s appearance in the set of matching genome sequences sets its probability of being selected in the next position. The selected base is added to the sequence and the n-gram window is shifted one step to the left and used to generate a new context for matching genomic sequences and a new set of per-base probabilities. The cycle repeats until the sequence reaches a desired length; here, all sequences were extended 35 bases upstream of the start codon.

The method is summarized in Fig 1. In any organism with a sequenced and annotated genome, we initialize our sequence generation with a two-base pair representing the second and third bases of the start codon (most often the TG of an ATG start codon, with rare exceptions). Querying all 5′‑UTRs for each annotated gene, we find all instances where this two-base sequence occurs, and determine the fraction of time each of the four possible bases occur in the 0 position (the first base of the start codon). A new base is chosen by generating a random number and selecting the bases with probabilities equal to their fractional representation in the genome. The “n-gram window” (equivalently, the context size) of two bases then takes one step upstream, identifying a new pair of bases and initiating a new query into the genome for the fractions of bases upstream of that pair, to be used for the weighted probabilities of each base occurring at the −1 position. Iterating the process of sliding the window upstream generates a sequence of bases with frequencies determined by the organism’s genome, but without any motif-based or mechanistic information explicitly incorporated into the selection; this is equivalent to the way machine learning-based language models step through sequences of words to predict or generate sentences. Resetting the process and generating a new set of pseudo-random numbers will yield a new sequence, and this process can be quickly repeated to generate a library of any desired size. (Generating 10,000 sequences took approximately 12 seconds on a single-core 3.4 GHz CPU.) It is possible to use an n-gram window with n greater than 2, but this comes with a clear trade-off between increasing contextual information (larger n values) and decreasing genomic information. Our *E. coli* reference genome provides 4449 known 5′‑UTR sequences, while our *L. reuteri* reference genome provides 1952 known sequences, and larger context sizes return smaller sets of matching sequences from the genome of interest. The average number of matching sequences from each organism’s genome is plotted as a function of the context size (n) in S2 Fig. For *E. coli*, the average number of matching genomic samples drops off as n increases: 1500 (n = 1), 375 (n = 2), 93 (n = 3), and 23 (n = 4). In *L. reuteri*, the average numbers of matching sequences found in the genome as a function of n were: 488 (n = 1), 122 (n = 2), 31 (n = 3), and 9 (n = 4). We wanted to obtain an average sample size of over 100 for both organisms, leading us to select n = 2 as the window size providing the best available trade-off between contextual information and the number of samples matching the context.

**Fig 2:**
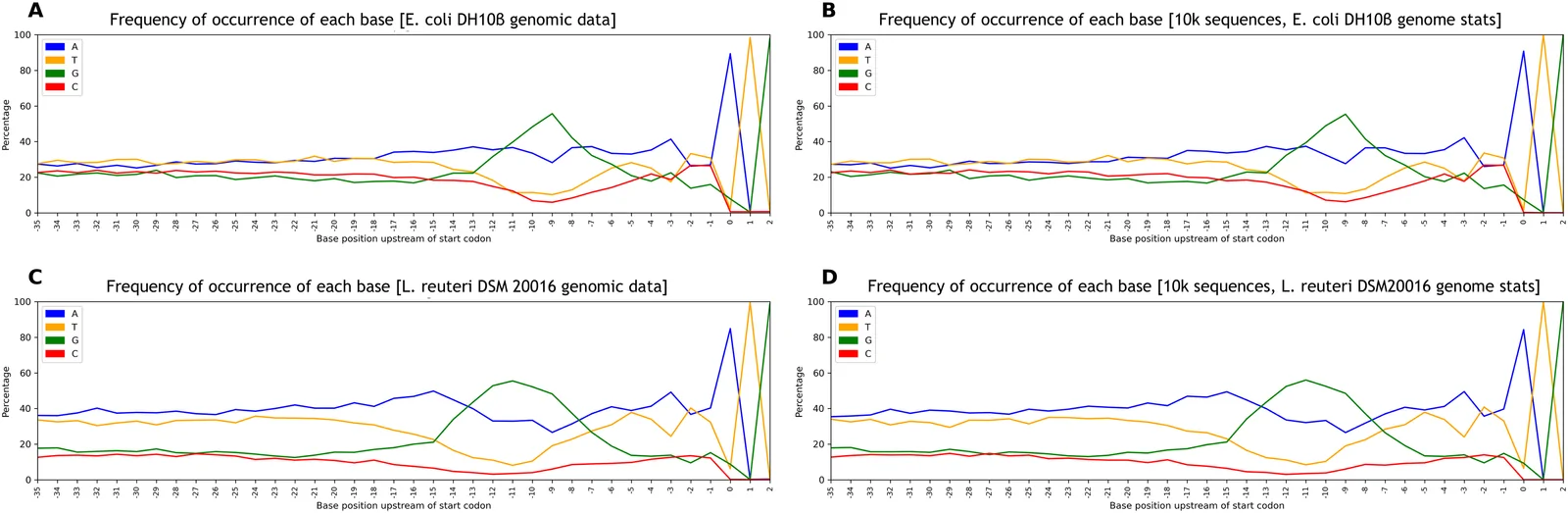
Single base frequencies in the 5′-UTR, in two organismal genomes and in 10,000 sequences generated by our algorithm, from the start codon to position −35 upstream. **(A, B)** The single base frequencies in the *E. coli* DH10β genome (A) and in our algorithmically generated set of sequences (B). **(C, D)** The single base frequencies in the *L. reuteri* DSM20016 genome (C) and in our algorithmically generated set of sequences (D). As expected, the algorithmic sequences retain nearly identical base frequencies, and include features such as the shifted location of a G‑rich region between the two organisms, and the overall higher occurrence of adenines in *L. reuteri*’s upstream region versus *E. coli*’s more even distribution among the four bases.

We have explored the range of translational efficiencies resulting from varying the 5′ untranslated regions in two bacterial species: *E. coli* (an extremely well−studied microbe); and *L. reuteri* DSM20016 (a non−model microbe that has been the subject of much less extensive study and characterization). Fig 2 shows the frequencies of single bases at each position from +2 to -35 upstream of the start codon, based on examining 4449 5′‑UTR sequences from the *E. coli* DH10β genome (NCBI accession number NC_010473), and examining 1952 5′‑UTR sequences from the significantly smaller *L. reuteri* DSM20016 genome (strain F275). At the right side of the plots, the dominant ATG start codon appears as near-100% frequencies for A, T, and G in positions 0, 1, and 2; this applies to both organisms, as expected. The enrichment of adenine (A) and guanine (G) bases in the -12 to -7 region in *E. coli* (Fig 2A) is consistent with the frequent inclusion of a Shine-Delgarno consensus sequence (AGGAGG) in that region. Results from the *L. reuteri* genome show a wider region of stronger G and A enrichment, located slightly further upstream than the equivalent region in *E. coli* (Fig 2C). The *L. reuteri* frequencies also indicate broad enrichment of adenine across the entire region upstream of the G- and A-enrichment site in line with the organism’s genome-wide low GC content of 38.9% [31,32], versus the *E. coli* genome’s more even distribution of base frequencies in the equivalent region, in line with its GC content of 50.8% [33].

Additional statistical tests were applied to the resulting sequences, including a calculation of the average information-theoretic entropy per base as a function of the context size (n): the entropy starts in the vicinity of 1.7 bits per base for n = 1, falling to near 0 bits by n = 10, with some variation between the two organisms (S3 Fig). To evaluate the degree of similarity between algorithmically generated sequences and sequences found in the two organisms’ genomes, we calculated minimum Hamming distances (S4 Fig). (The Hamming distance between two sequences assigns a 1 to identical bases and a 0 otherwise.) As expected, small context sizes (n = 1 to n = 3) showed substantial differences, with the bulk of the generated sequences differing by a minimum of 12–18 bases from the set of genomic sequences. At larger context sizes (n = 6 to n = 10), much larger fractions of the generated sequences were nearly identical to existing sequences in the genome. By n = 10, about 71% of the generated sequences in *E. coli* were identical to a sequence in the genome, while about 57% of the generated sequences in *L. reuteri* were identical to an existing genomic sequence. We generated a theoretical estimate of the rate at which the algorithm generates duplicate sequences, as a function of the context size, then computationally generated sets of sequences in which the duplication rates matched the predictions (S5A, S5B Fig). For low context sizes (n < 5), the expected numbers of sequences before encountering a repeat are in the tens of millions to the low billions, indicating that generating duplicate sequences is unlikely for moderate library sizes. At the highest context sizes, the expected number of sequences before encountering a duplicate converges to a limit defined by the total number of sequences in the genome (~84 for the *E. coli* generated sequences, and ~55 for the *L. reuteri* generated sequences). At these higher context sizes, duplicated sequences would be expected to occur regularly, even in small libraries.

### *in cellulo* testing

We used the approach shown in Fig 1, with n = 2, to randomly generate 1000 5′‑UTR sequences derived from the genomic statistics of *E. coli* DH10β and *L. reuteri* DSM20016. After plasmid assembly and quality control to exclude errors in the assembly process (see Methods) we were left with over a hundred verified sequences drawn from each organism’s genomic information, and we experimentally tested each sequence in both organisms, finding a wide range of translation levels even in this small library and noting interesting organism-specific properties of the sets of candidate sequences.

5′‑UTR sequences generated by the Fig 1 algorithm based on both *E. coli* and *L. reuteri* genomic statistics were incorporated into otherwise identical PTRKH3 plasmid backbones, and transformed into *E. coli*. 176 colonies from each 5′‑UTR pool were picked at random, grown and tested in *E. coli* for activity, and sent for sequencing. After removing duplicate/triplicate 5′‑UTR sequences that arose during the assembly process, 123 unique *E. coli* sequences and 117 unique *L. reuteri* sequences remained. These were then transformed into *L. reuteri* DSM 116333 (a naturally derived sub-strain of DSM 20016 with higher transformation efficiency and more stable reporter protein production) [29,30] and tested for activity in the form of fluorescent reporter protein expression. The full sequences lists and experimental measurements are provided as CSV files in S1 Table (*E. coli*-derived sequences) and S2 Table (*L. reuteri*-derived sequences).

For purposes of tuning the expression level of a protein of interest, we consider a wide, uniform distribution to be the goal: a set of candidate 5′‑UTR sequences should span a wide range of expression levels, and in the ideal case the sequences would be uniformly distributed over the full range. A perfectly uniform distribution of expression levels would enable optimal expression tuning, allowing one to select a sequence to provide an expression level at any point in the full range. To quantify the degree of uniformity of the experimental distributions, we calculated the normalized Shannon entropy (H_norm_) of each observed distribution, a metric that varies from 1.0 for a perfectly uniform distribution to 0.0 for a “distribution” in which all sequences have the same expression value (see Methods).

**Fig 3:**
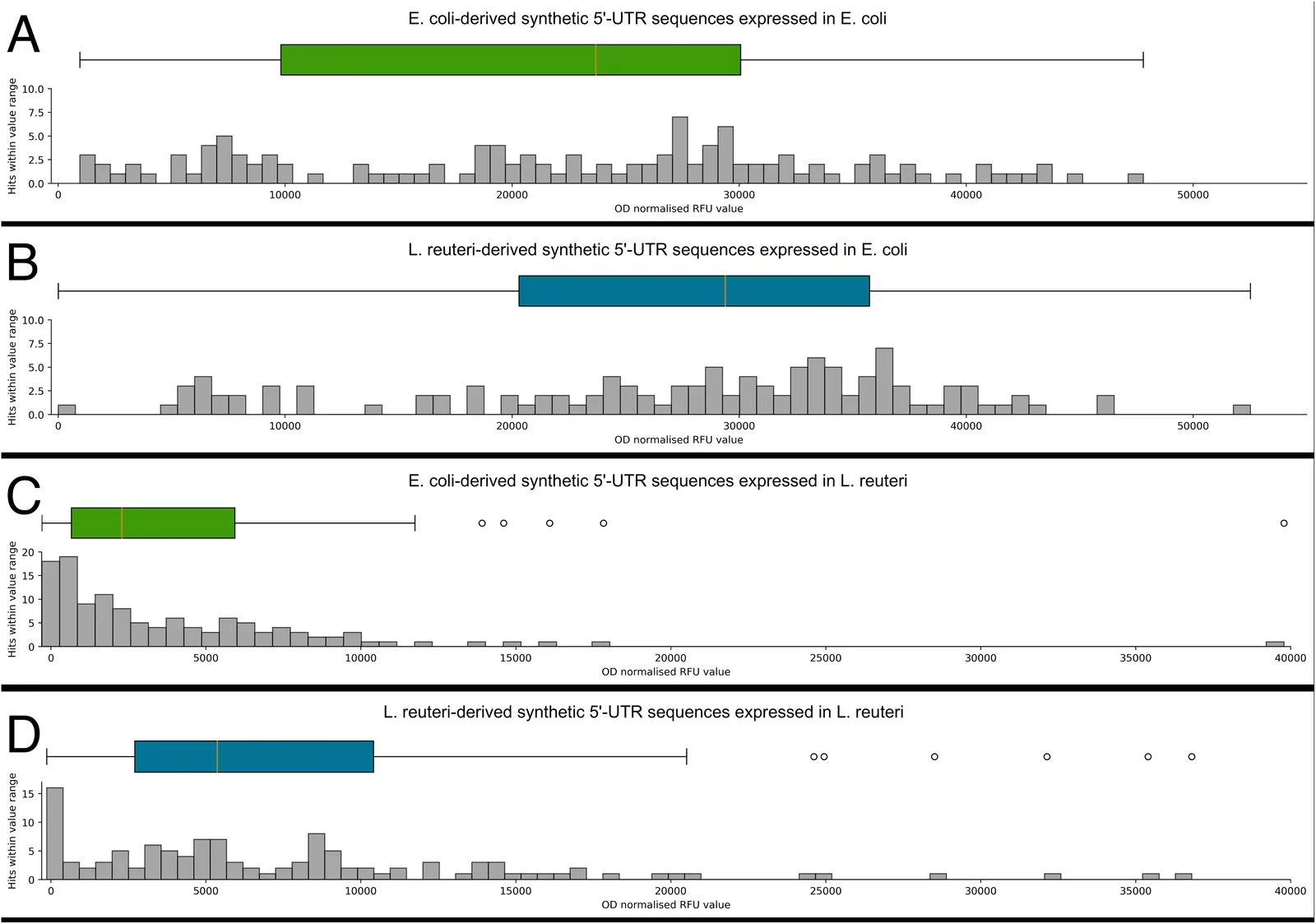
A) *E. coli* derived synthetic 5′-UTR sequences transformed into *E. coli* DH10β. B) *L. reuteri* derived synthetic 5′ UTR sequences transformed into *E. coli* DH10β C) The same *E. coli* derived 5′ UTR sequences from panel A, transformed into *L. reuteri* DSM 116333. D) The same *L. reuteri* derived synthetic 5′ UTR sequences from panel B, transformed into *L. reuteri* DSM 116333. One replicate for each condition.

None of the sequence pools achieved perfect uniformity, but some trends are notable. In *E. coli*, the sequences derived from *E. coli*’s own genomic data were somewhat more uniform (H_norm_ of 0.88) than the sequences derived from *L. reuteri*’s genomic data (H_norm_ of 0.83) (Fig 3A, 3B). In *L. reuteri*, the *E. coli*-derived 5′‑UTR sequence pool had a lower uniformity score (H_norm_ of 0.67) than the *L. reuteri*-derived sequence pool (H_norm_ of 0.78). This difference is visible by eye in the distributions, with the *E. coli*-derived sequences noticeably more clustered at the low end of the distribution and only sparsely distributed over the higher end of the range of expression levels (Fig 3C, 3D). The differential uniformity scores suggest that for purposes of expression tuning, using sequences derived from each organism’s own genomic data offers a wider range of options, with the improvement in uniformity being more substantial in *L. reuteri* than in *E. coli*. It is not clear at this stage whether such improvements will generalize to all organisms, but these initial results suggest that further investigation in a wider set of organisms could be productive.

It is somewhat surprising that the *L. reuteri*-derived sequences provided a higher average expression level in *E. coli* than the native *E. coli*-derived sequences did (Fig 3B vs Fig 3A). Our method does not provide an explanation for this phenomenon since it does not incorporate mechanistic elements into the sequence generation process. We note, however, that the native upstream sequences in a given organism have not necessarily evolved to *maximize* protein expression levels, but rather to *optimize* them in the full organismal context, and it is not clear that the optimal native expression levels in an organism must necessarily be the highest ones. It is possible that non-native upstream sequences could shift expression to levels higher than an organism’s native average levels.

Resource constraints have precluded us from carrying out side-by-side comparisons with pools of fully randomly generated sequences, but we note that the fully random sequences tested in a previously published set of experiments [29] show a set of translational levels that is strongly clustered near the low end of its range. The same resource constraints have constrained our efforts to experimentally explore n = 1 or n = 3 context sizes, but future explorations of the approach could explore a wider range of context sizes and random-sequence variants.

**Fig 4:**
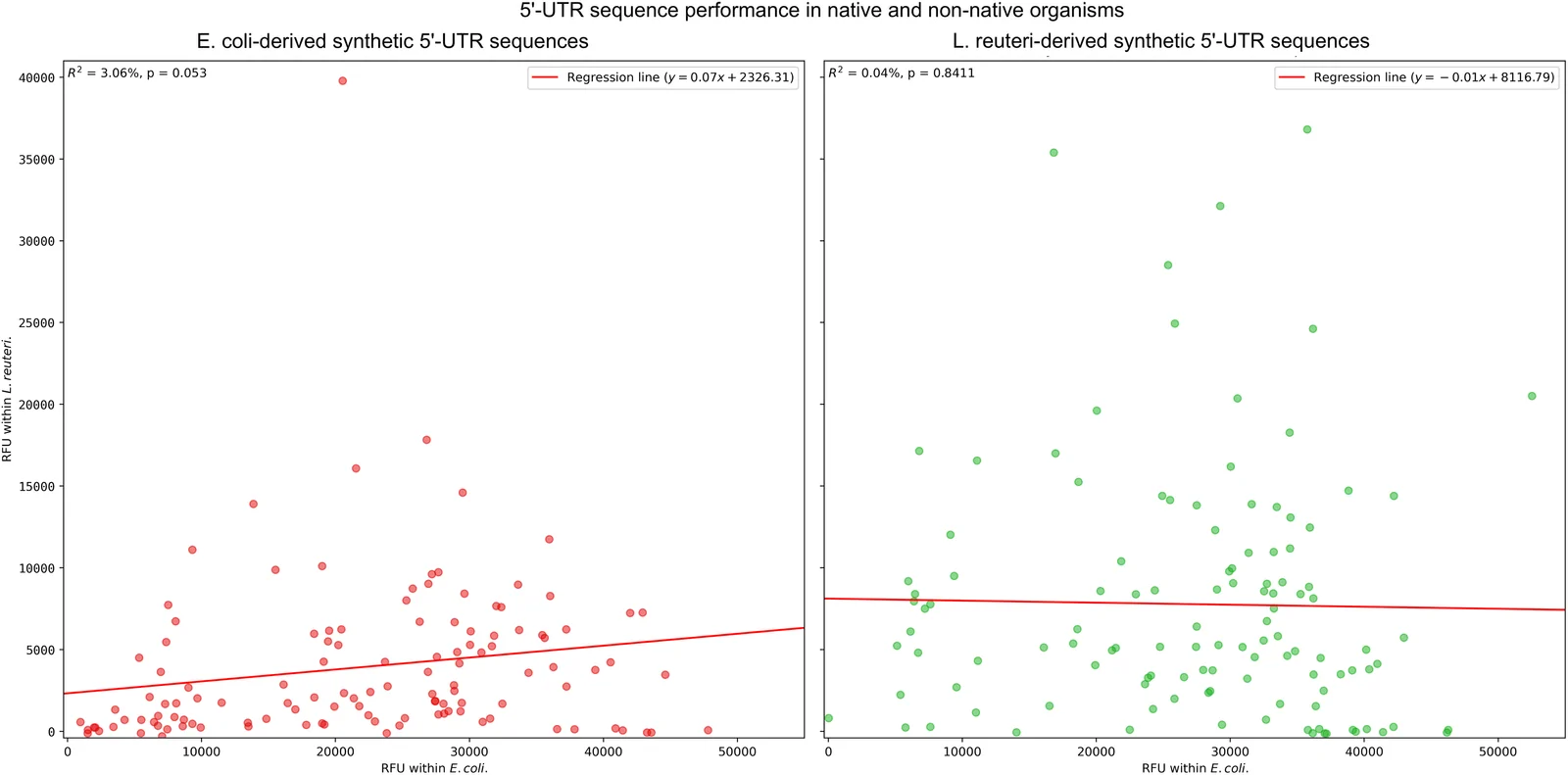
Strengths of our generated 5′-UTR sequences, experimentally measured with fluorescent reporter protein production, in two bacterial species. In each plot, the strength of an individual sequence in *E. coli* is plotted on the horizontal axis, while the strength of the same sequence in *L. reuteri* is plotted on the vertical axis. (left) Strengths of 5′-UTR sequences derived from the *E. coli* genome, in the two species; (right) Strengths of RBS sequences derived from the *L. reuteri* genome, in the two species. RFU: relative fluorescence units. One replicate for each condition.

Testing every sequence in both bacterial species enabled us to examine cross-species correlations: to what extent did high/low performance of a given sequence in one organism correlate with similarly high/low performance in the other? Fig 4 plots the activity of each sequence in *L. reuteri* against the activity of the same sequence in *E. coli*, for the *E. coli*-derived sequences and for the *L. reuteri*-derived sequences. In both cases, the conclusion is both visually and mathematically clear: the correlations are weak. The *E. coli*-derived sequences appear to show a very slight positive trend (Fig 4A), but the R^2^ value of 0.03 makes it clear that noise dominates. *L. reuteri*-derived sequences show a “trend” line that is nearly flat (Fig 4B), and the R^2^ value of 4x10^‑4^ makes it clear that there is essentially no relationship between how a given sequence from that pool will perform in the two organisms.

**Fig 5:**
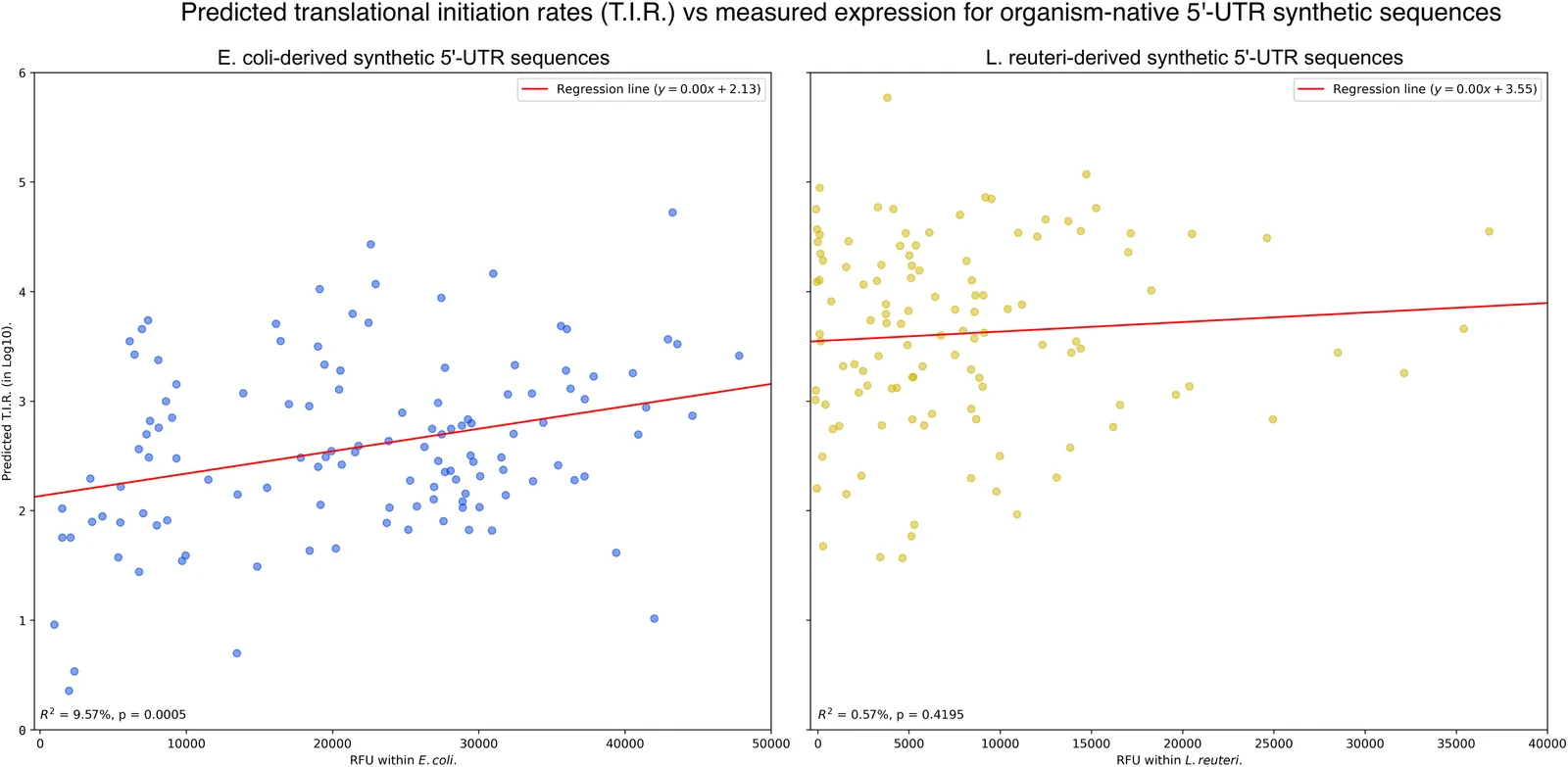
Predicted strengths of our generated 5′-UTR sequences [14,33,34,36] compared to strengths experimentally measured with fluorescent reporter protein production, in two bacterial species. In each plot, the predicted translational initiation rate (T.I.R.) of sequences derived from the genome of each species is plotted (log_10_ scale) on the vertical axis, against the experimentally measured expression level on the horizontal axis. (left) Predicted strengths of 5′-UTR sequences derived from the *E. coli* genome vs measured strengths in *E. coli*. (right) Predicted strengths of 5′-UTR sequences derived from the *L. reuteri* genome vs measured strengths in *L. reuteri*. RFU: relative fluorescence units. One replicate for each condition.

Tools are available to generate predictions of binding strengths between ribosomal 16S subunits and target mRNA sequences and the resulting translational initiation rates, and we have used one well-established tool of this type [14,34,35] to compare these predicted binding strength values for each 5′-UTR sequence in each of our two organism-specific pools to our experimental observations for the corresponding sequence in each organism. Fig 5 shows the result of plotting these predictions against the observed activity in each organism. In *E. coli* (Fig 5A), there is a weak but noticeably positive trend with an R^2^ value of 0.096. In *L. reuteri* (Fig 5B), once again the “trend” line is nearly flat, with the R^2^ value of 5.7x10^-4^ confirming that there is essentially no correlation between the predicted binding strengths and our observed activity levels in the non-model organism.

We examined the sets of experimentally tested sequences to attempt to identify any regularities between the sequences and their measured expression levels. Since *E. coli* and *L. reuteri* share the same Shine-Dalgarno consensus (AGGAGG), we searched the 18 bases upstream of the start codon (considered to be the most likely location range for the S-D sequence) for occurrences of this sequence, scoring each experimental sequence with the minimum Hamming distance between the S‑D sequence and any 6-base subset; a score of 0 indicates that the exact S‑D consensus sequence occurred at least once, while higher scores indicated the presence of sub-sequences with 1, 2, 3, or 4 differing bases (S6 Fig). The results showed a very slight negative trend with increasing Hamming distance from the S‑D consensus, but the R^2^ value of 0.045 indicated that the correlation was quite weak. We used the open-source ViennaRNA library (github.com/ViennaRNA) to predict the minimum folding free energy of the RNA sequences associated with each experimentally tested sequence and plotted the measured translation strengths against these folding energies (S7 Fig). The results show essentially no correlation between the predicted folding free energies and the measured translational strengths of the sequences. A search for correlations between k-mer motifs for k = 4 to k = 6 using the Lasso method implemented in the scipy Python package (LassoCV) failed to show any statistically convincing correlations between individual 4- to 6-base motifs and the measured strengths of our experimentally tested sequences (S3 Table); this may be mainly a sample size limitation imposed by the need to parameterize models of size ${\text{4}}^{\text{4}}=\text{2}\text{5}\text{6}$ (for 4-base k-mers), ${\text{4}}^{\text{5}}=\text{1}\text{0}\text{2}\text{4}$ (5-base), and ${\text{4}}^{\text{6}}=\text{4}\text{0}\text{9}\text{6}$ (6-base) using only ~120 experimental measurements.

Figs 3–5 combine to tell a clear story: the model organism *E. coli* is easier to work with across the board than the non-model *L. reuteri*, showing a very uniform range of translational activity from 5′-UTR sequences derived from its own genomic statistics, and retaining a slightly reduced but still high uniformity in the activities of 5′‑UTR sequences derived from the genomic statistics of *L. reuteri*. Individual sequence results from the *E. coli* genome correlated slightly better across the two organisms, and were somewhat more predictable from binding strength estimates. This level of ease of use and predictability is, presumably, correlated with *E. coli*’s status as a highly favoured and widely adopted model organism. *L. reuteri* was generally more difficult to work with: the tested 5′-UTR sequences showed lower overall activity levels and less uniformity in the distributions, though it is promising that both the range of activity levels and the uniformity of the distribution improved when using sequences generated from *L. reuteri*’s own genome. The sequences generated using *L. reuteri* genome offered fewer sequence options to cover the range of activities, showed very low correlations in sequence performance across the two organisms, and displayed effectively no correlation between predicted binding strengths and experimental activity levels.

The simplicity of our approach neglects long-range influences in the genomic sequence space and does not bring to bear cutting-edge technologies like machine learning approaches or other sophisticated methods for the analysis of large data sets such as complete organismal genome sequences. We would note, however, that this simplicity has its own positive features: the computational demands are minimal, the success rate is high enough that screening even small libraries is likely to yield sequences offering a range of expression levels, and switching between organisms is a simple matter of priming the algorithm with a different genomic sequence. These features position the approach to be widely accessible, lowering the barriers that might otherwise hamper investigations in non-model organisms. Though our approach worked better in the model organism we tested, it is less critical in that context: when working in *E. coli*, there is no shortage of well-validated methods available for tuning gene expression at every level, whereas work in *L. reuteri* and other non-model organisms may suffer from a distinct lack of equivalent options.

The results we have obtained here illustrate that even a simplified language model based on a “sliding window” approach to deriving sequence statistics from genomic information can generate a wide range of translation levels from a tractable library size. Further development of the approach could involve its application to new organisms (particularly to eukaryotes, to determine whether the method can be extended to that context), and to new classes of sequences, such as promoters or origins of replication.

## Methods

### Sequence generation

The code for the sequence generation algorithm is available at: https://github.com/AIex-Duggan/RBS-Language-Model.

5′-UTR sequences were extracted 35 bases upstream of the start codon (including putative start codons) and aligned to the start codon site. The algorithm begins with a 38 base sequence in which any base is possible at any position. The model for this project was fixed to start with the ‘TG’ codons of the regular and alternative start codons and allowed to deduce next positions from that initial seed, extending the sequence from right to left (equivalently: from downstream to upstream, or from the 3′ to 5′ end of the sequence). The model utilises a simple sliding window n-gram design, with n = 2 in our current implementation. At each position, the next base in the sequence is selected at random, with the selection probability for each base determined by its frequency in the set of matching sequences from the target organism’s genome, where a genomic sequence matches if it has the same two bases in the equivalent positions relative to the start codon. The probabilities are given by


$$\begin{array}{c} P\left(c_{k}|c_{k-\text{1}:k-\text{2}}\right)=\frac{N\left(c_{k}|c_{k-\text{1}:k-\text{2}}\right)}{N(c_{k-\text{1}:k-\text{2}})}\;, \end{array}$$

where at position *k*, the probability, *P*, of selecting each base [*c*_*k*_] is set to the fraction of genomic sequences in which it appears (the number, *N*, of such sequences, divided by the total number of matching sequences obtained from the organism’s genome).

### Plasmid construction

Plasmid pTRKH3_mCherry2 (first developed in previous work [29,30] was modified with standard PCR to remove the “universal RBS” BBa_K2918014 sequence, as well as removing the start codon for mCherry2.

Synthetic 5′-UTR sequences generated by the algorithm were augmented at the point of synthesis with additional bases at both ends containing Golden Gate restriction sites to allow scarless integration into a linearized backbone and primer sites to allow PCR amplification. Oligonucleotides were obtained in two batches (one for each organism) containing the full set of DNA sequences in a single-tube mixed pool (Twist Biosciences). Each oligonucleotide pool was subjected to PCR to convert from ssDNA to dsDNA, following a PCR protocol modified from manufacturer recommendations (Twist Biosciences), replacing the recommended KAPA polymerase with Q5 polymerase for improved sequence fidelity. Golden Gate assembly was used to assemble the synthetic 5′-UTR sequences and linear PCR products obtained from the plasmid backbone, and ligation of the final product into the original pTRKH3 backbone placed the 5′-UTR sequences upstream of the mCherry2 gene. See S1 Fig in the Supplementary Information for primer schematics and complete sequences.

### Cell handling and measurement

The two plasmid pools (with synthetic 5′-UTR sequences derived from *E. coli* and from *L. reuteri*) were each transformed into chemically competent *E. coli* DH10β and plated on LB-agar for isolation. The desired size of the pool of correctly assembled plasmids to be experimentally tested needed to balance statistical considerations (the more samples the better) against practical considerations (the non-trivial challenge of extracting, sequencing, measuring, and individually storing/tracking each plasmid). A target of ~100 sequenced and verified plasmids per organism-derived pool was judged to be large enough to provide an informative sample size, while remaining experimentally tractable. To allow for attrition during the verification of correct assembly, 176 colonies were isolated from each condition (an arbitrary choice made during the colony-picking step), grown in 6 ml LB broth with appropriate selection antibiotic, subject to plasmid extraction (QIAprep mini-prep, Qiagen Canada). All plasmids were sent for sequencing with primers designed to capture the full region of the synthetic sequences and the start of the mCherry2 gene.

Assembled plasmid candidates from both organism-derived sequence pools were then independently subjected to several forms quality control based on the results of sequencing the variable regions of the assembled plasmids, eliminating plasmids based on several indicators of probable failures of correct plasmid assembly: missing or incomplete UTR sequences (indicating ligation failures); duplicated sequences (indicating multiple ligations); poor sequencing results (eliminated to avoid including plasmids with uncertain sequences); and contamination of samples with multiple plasmids (indicated by the presence of multiple visible peaks on sequencing chromatograms). After quality control, 123 *E. coli* RBS sequences and 117 *L. reuteri* sequences were retained as the final pools for each organism-derived set of sequences, and transformed into both chemically competent *E. coli* DH10β and electrocompetent *L. reuteri* DSM116333 [29].

*E. coli* DH10β was grown in LB broth (BioShop, Canada) aerobically at 37°C with shaking at 200RPM, LB agar was grown aerobically at 37°C for 16–24 hours. Erythromycin was used to maintain pTRKH3 plasmid selection at 250 µg/ml in both broth and agar.

*L. reuteri* DSM116333 was grown aerobically in autoclaved MRS broth (Oxoid, UK) at 37°C with no shaking, MRS agar was grown anaerobically at 37°C for 24–48 hours or until colonies were visible. Erythromycin was used to maintain pTRKH3 plasmid selection at 10 µg/ml in both broth and agar.

For fluorometric assays *E. coli* was grown in LB broth (conditions as above) with selective antibiotic, and after 24 hours 200 µl aliquots were transferred to a flat-bottom optical 96-well plates for OD600 and mCherry2 production measurement (Ex:589 Em:610). *L. reuteri* was grown for 24 hours in filter sterilised MRS broth (conditions as above), and 200 µl aliquots were measured with the same Ex:Em parameters.

Each set of fluorometric assays (Figs 3–5) reports a single experimental replicate, rather than the more standard three replicates. A dropped plate after the first experimental run caused the samples to mix in unknown ways across the plate, making the planned additional runs impossible without ordering a full new set of sequences for each organism and re-implementing the plasmid assembling, sequence, and quality control process. The cost of this process was beyond the scope of available funding, so we are presenting the single-replicate runs available; this prevents us from reporting run-to-run variations, but does still offer useful information about the behaviour of the sequences.

### Uniformity metric for experimental distributions

Having identified a perfectly uniform distribution of translation strengths as the ideal result for tuning purposes, we calculated the normalized Shannon entropy as a quantitative metric of the degree of uniformity of each of our four experimental distributions (Fig 3). For each distribution, the range of activity was separated into N equal-sized bins from zero to the maximum reported value, where N was the number of sequences tested. For a perfectly uniform distribution, one would have exactly one sequence per bin. We computed the Shannon entropy based on the fraction of 5′-UTRs in each bin ($p_{i}$), summing over all bins to find the Shannon entropy across the distribution (using the limit $\underset{p_{i}\to \text{0}}{\text{lim}}p_{i}\text{ln}(p_{i})=\text{0}$ for cases with $p_{i}=\text{0}$), then normalized by the maximum possible entropy ($H_{max}=\text{ln}N$, obtained when $p_{i}=\frac{\text{1}}{N}$ for all i) to obtain a value between 0 and 1:


$$\begin{array}{c} H_{norm}\;=\;\frac{\sum_{i=\text{1}}^{N}\left(-p_{i}\text{ln}(p_{i})\right)}{\text{ln}(N)} \end{array}$$

## Supporting information

S1 Fig Synthetic 5′-UTR sequence primers for Golden Gate assembly. (PNG)

S2 Fig Number of samples returned from each organism’s genome, as a function of the context size (n). The average number of matching sequences returned from the genome for context sizes varying from n = 1 to n = 10. Starting at the −11 upstream position (the first position for which the n = 10 context size does not overlap with the start codon), the number of genomic 5′-UTR sequences matching all permutations of sequences with each context length were recorded and averaged to obtain the means shown as dots. The bars indicate the maximum and minimum number of sequences obtained at each context size.
(PNG)

S3 FigAverage Shannon entropy per base for varying context sizes, n, in the n-gram sliding window algorithm.The entropy (H, in bits per base) for each value of n was calculated as $H=-\frac{\text{1}}{N}\sum_{i}{\text{log}}_{\text{2}}P(x_{i}|\text{context})$ where the context is the previous *n* bases in a given genomic 5′-UTR sequence, $x_{i}$ is the next base in the 5′-UTR sequence, and N is the total number of contexts examined across a genome. For evaluating the average entropy for a genomic 5’ UTR context model, contexts smaller than *n* bases upstream are ignored (for example, the context “*TG*” within the start codon is ignored for *n*-gram models of size three or higher), to maintain size uniformity across all context lengths.(PNG)

S4 Fig Minimum Hamming distances between algorithmically generated sequences and native genomic sequences, as a function of context size (n). Each generated sequence was compared to all genomic sequences, a Hamming distance was calculated (assigning a 1 for a matching base in a given position, and a 0 otherwise), and the minimum Hamming distance across all such comparisons was recorded as the result for that sequence. The histograms show the result of carrying out this operation on 100,000 sequences generated independently for each organism at each context size. Histograms were generated for all context sizes from n = 1 to n = 10, but for clarity only the three lowest (n = 1 to n = 3) and a selection of the highest (n = 6, 8, 10) are shown here; the distributions shift gradually to the left as n increases from 4 to 10.
(PNG)

S5 Fig Distributions of number of algorithmically generated sequences before a repeated sequence. A) Sequences derived from the *E. coli* genome. B) Sequences derived from the *L. reuteri* genome. Black dots and violin plots give the number and distribution, respectively, of sequences generated before encountering a repeat, in computational simulations at context sizes n = 5 to n = 10. (Lower context sizes required unfeasibly large numbers of sequences to be generated and pairwise compared, so simulations were not conducted for n = 1 to n = 4.) The expected number of sequences before repetition can be estimated as a function of n, and these estimates are plotted as the red horizontal dashed lines at each value of n; details of that calculation are provided below. Black horizontal dashed lines: an approximate lower bound on the number of sequences between repeats, defined by the number of genomic sequences.
(PNG)

S6 Fig Minimum Hamming distance to the consensus Shine-Dalgarno sequence. The first 18 bases of the experimentally tested sequences generated with each organism’s genomic data were evaluated for the presence of the AGGAGG sequence, which is the consensus S-D sequence in both organisms. The AGGAGG sequence was compared against every contiguous 6-base sequence in the first 18 bases upstream of the start codon: a Hamming distance was assigned at every position within that range, and the minimum of all Hamming distances recorded as the result for each sequence. A value of 0 indicates at least one exact occurrence of the S-D consensus sequence somewhere in the range, with higher values indicating the occurrence of sequences with 1, 2, 3, or 4 based differing from the S-D consensus. We have plotted the experimental expression strengths (measured by OD-normalized fluorescence) as a function of this minimum Hamming distance. Green dots: sequences generated from *E. coli* genomic data. Brown dots: sequences generated from *L. reuteri* genomic data. Dashed black line: linear regression using all data points (not separated by organism since they share an S-D consensus sequence). Numbers at the top indicate the number of experimental sequences in each category.
(PNG)

S7 Fig Expression level (measured by OD-normalized fluorescence) as a function of predicted folding free energies. The minimum free energy associated with folding of the RNA sequences corresponding to each of the experimentally tested sequences was generatd using the ViennaRNA open source library (github.com/ViennaRNA). Green dots: sequences generated from *E. coli* genomic data. Brown dots: sequences generated from *L. reuteri* genomic data. Dashed black lines: linear regression using all data points from both organisms (since RNA folding energies should depend only on the sequence itself, not how it was originally generated).
(PNG)

S1 Table A spreadsheet in CSV format (file downloadable separately), providing the raw data for the experimental measurements of each of 123 sequences generated using genomic data from *E. coli.* The sheet provides the measured fluorescence from each sequence when a plasmid containing the sequence was transformed into *E. coli* and when the same plasmid was transformed into *L. reuteri.*
(CSV)

S2 Table A spreadsheet in CSV format (file downloadable separately), providing the raw data for the experimental measurements of each of 117 sequences generated using genomic data from *L. reuteri.* The sheet provides the measured fluorescence from each sequence when a plasmid containing the sequence was transformed into *E. coli* and when the same plasmid was transformed into *L. reuteri.*
(CSV)

S3 Table A spreadsheet in CSV format (file downloadable separately), providing the results of applying a Lasso k-mer motif analysis to our experimentally tested sequences. (CSV)
